# Unsupervised Feature Learning Improves Prediction of Human Brain Activity in Response to Natural Images

**DOI:** 10.1371/journal.pcbi.1003724

**Published:** 2014-08-07

**Authors:** Umut Güçlü, Marcel A. J. van Gerven

**Affiliations:** Radboud University Nijmegen, Donders Institute for Brain, Cognition and Behaviour, Nijmegen, Netherlands; Medical Research Council, United Kingdom

## Abstract

Encoding and decoding in functional magnetic resonance imaging has recently emerged as an area of research to noninvasively characterize the relationship between stimulus features and human brain activity. To overcome the challenge of formalizing what stimulus features should modulate single voxel responses, we introduce a general approach for making directly testable predictions of single voxel responses to statistically adapted representations of ecologically valid stimuli. These representations are learned from unlabeled data without supervision. Our approach is validated using a parsimonious computational model of (i) how early visual cortical representations are adapted to statistical regularities in natural images and (ii) how populations of these representations are pooled by single voxels. This computational model is used to predict single voxel responses to natural images and identify natural images from stimulus-evoked multiple voxel responses. We show that statistically adapted low-level sparse and invariant representations of natural images better span the space of early visual cortical representations and can be more effectively exploited in stimulus identification than hand-designed Gabor wavelets. Our results demonstrate the potential of our approach to better probe unknown cortical representations.

## Author Summary

An important but difficult problem in contemporary cognitive neuroscience is to find what stimulus features best drive responses in the human brain. The conventional approach to solve this problem is to use descriptive encoding models that predict responses to stimulus features that are known a priori. In this study, we introduce an alternative to this approach that is independent of a priori knowledge. Instead, we use a normative encoding model that predicts responses to stimulus features that are learned from unlabeled data. We show that this normative encoding model learns sparse, topographic and invariant stimulus features from tens of thousands of grayscale natural image patches without supervision, and reproduces the population behavior of simple and complex cells. We find that these stimulus features significantly better drive blood-oxygen-level dependent hemodynamic responses in early visual areas than Gabor wavelets–the fundamental building blocks of the conventional approach. Our approach will improve our understanding of how sensory information is represented beyond early visual areas since it can theoretically find what stimulus features best drive responses in other sensory areas.

## Introduction

An important goal of contemporary cognitive neuroscience is to characterize the relationship between stimulus features and human brain activity. This relationship can be studied from two distinct but complementary perspectives of encoding and decoding [1]. The encoding perspective is concerned with how certain aspects of the environment are stored in the brain and uses models that predict brain activity in response to certain stimulus features. Conversely, the decoding perspective uses models that predict specific stimulus features from stimulus-evoked brain activity and is concerned with how specific aspects of the environment are retrieved from the brain.

Stimulus-response relationships have been extensively studied in computational neuroscience to understand the information contained in individual or ensemble neuronal responses, based on different coding schemes [2]. The invasive nature of the measurement techniques of these studies has restricted human subjects to particular patient populations [3], [4]. However, with the advent of functional magnetic resonance imaging (fMRI), encoding and decoding in fMRI has made it possible to noninvasively characterize the relationship between stimulus features and human brain activity via localized changes in blood-oxygen-level dependent (BOLD) hemodynamic responses to sensory or cognitive stimulation [5].

Encoding models that predict single voxel responses to certain stimulus features typically comprise two main components. The first component is a (non)linear transformation from a stimulus space to a feature space. The second component is a (non)linear transformation from the feature space to a voxel space. Encoding models can be used to test alternative hypotheses about what a voxel represents since any encoding model embodies a specific hypothesis about what stimulus features modulate the response of the voxel [5]. Furthermore, encoding models can be converted to decoding models that predict specific stimulus features from stimulus-evoked multiple voxel responses. In particular, decoding models can be used to determine the specific class from which the stimulus was drawn (i.e. classification) [6], [7], identify the correct stimulus from a set of novel stimuli (i.e. identification) [8], [9] or create a literal picture of the stimulus (i.e. reconstruction) [10]–[12].

The conventional approach to encoding and decoding makes use of feature spaces that are typically hand-designed by theorists or experimentalists [8], [9], [11], [13]–[16]. However, this approach is prone to the influence of subjective biases and restricted to a priori hypotheses. As a result, it severely restricts the scope of alternative hypotheses that can be formulated about what a voxel represents. This restriction is evident by a paucity of models that adequately characterize extrastriate visual cortical voxels.

A recent trend in models of visual population codes has been the adoption of natural images for the characterization of voxels that respond to visual stimulation [8], [13]. The motivation behind this trend is that natural images admit multiple feature spaces such as low-level edges, mid-level edge junctions, high-level object parts and complete objects that can modulate single voxel responses [5]. Implicit about this motivation is the assumption that the brain is adapted to the statistical regularities in the environment [17] such as those in natural images [18], [19]. At the same time, recent developments in theoretical neuroscience and machine learning have shown that normative and predictive models of natural image statistics learn statistically adapted representations of natural images. As a result, they predict statistically adapted visual cortical representations, based on different coding principles. Some of these predictions have been shown to be similar to what is found in the primary visual cortex such as topographically organized simple and complex cell receptive fields [20].

**Figure 1:**
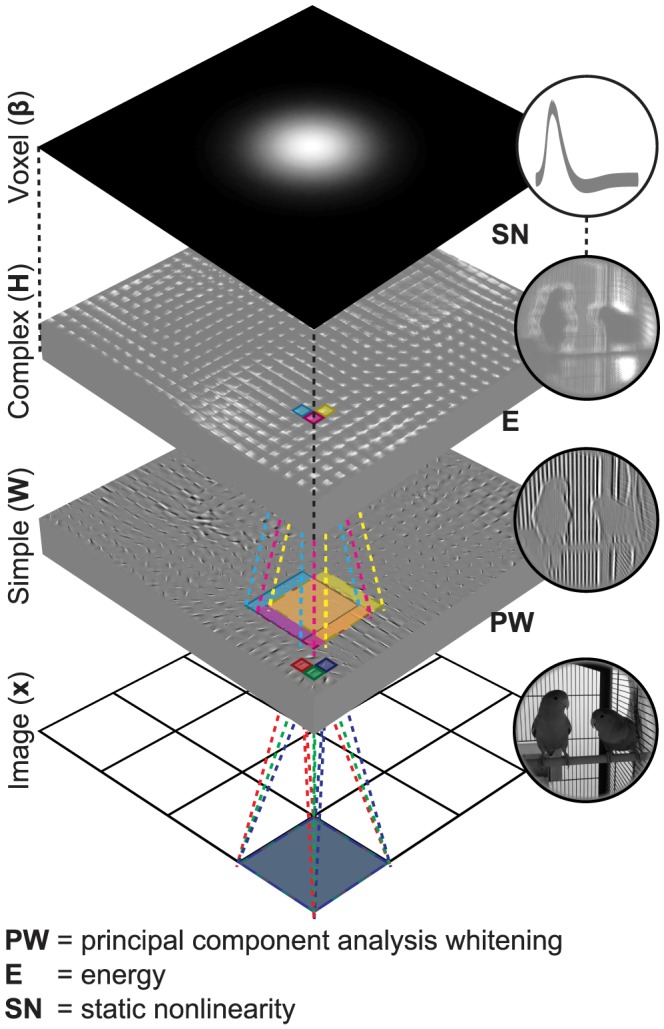
Encoding model. The encoding model predicts single voxel responses to images by nonlinearly transforming the images to complex cell responses and linearly transforming the complex cell responses to the single voxel responses. For example, the encoding model predicts a voxel response to a 128
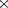
128 image 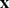 as follows: Each of the 16 non-overlapping 32
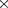
32 patches of the image 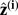 is first vectorized, preprocessed and linearly transformed to 625 simple cell responses, i.e. 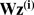 where 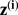 is a vectorized and preprocessed patch. Energies of the simple cells that are in each of the 625 partially overlapping 5
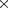
5 neighborhoods are then locally pooled, i.e. 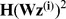, and nonlinearly transformed to one complex cell response, i.e. 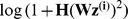. Next, 10000 complex cell responses are linearly transformed to the voxel response, i.e. 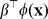 where 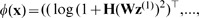 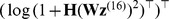. The feature transformations are learned from unlabeled data. The voxel transformations are learned from feature-transformed stimulus-response pairs.

Building on previous studies of visual population codes and natural image statistics, we introduce a general approach for making directly testable predictions of single voxel responses to statistically adapted representations of ecologically valid stimuli. To validate our approach, we use a parsimonious computational model that comprises two main components (Figure 1). The first component is a nonlinear feature model that transforms raw stimuli to stimulus features. In particular, the feature model learns the transformation from unlabeled data without supervision. The second component is a linear voxel model that transforms the stimulus features to voxel responses. We use an fMRI data set of voxel responses to natural images that were acquired from the early visual areas (i.e. V1, V2 and V3) of two subjects (i.e. S1 and S2) [21]. We show that the encoding and decoding performance of this computational model is significantly better than that of a hand-designed Gabor wavelet pyramid (GWP) model of phase-invariant complex cells. The software that implements our approach is provided at http://www.ccnlab.net/research/.

## Results

### Feature models

**Figure 2:**
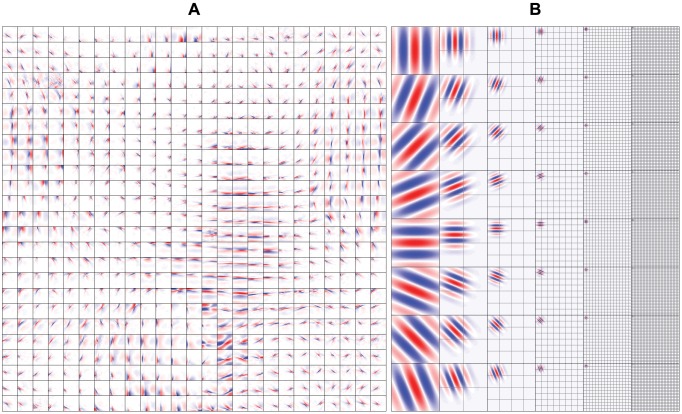
Simple cell receptive fields. (A) Simple cell receptive fields of the SC model. Each square is of size 32
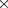
32 pixels and shows the inverse weights between the input and a simple cell. The receptive fields were topographically organized, spatially localized, oriented and bandpass, similar to those found in the primary visual cortex. (B) Simple cell receptive fields of the GWP model. Each square is of size 128
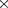
128 pixels and shows an even-symmetric Gabor wavelet. The grids show the locations of the remaining Gabor wavelets that were used. The receptive fields spanned eight orientations and six spatial frequencies.

To learn the feature transformation, we used a two-layer sparse coding (SC) model of 625 simple (i.e. first layer) and 625 complex (i.e. second layer) cells [22]. Concretely, the simple cells were first arranged on a square grid graph that had circular boundary conditions. The weights between the simple and complex cells were then fixed such that each complex cell locally pooled the energies of 25 simple cells in a 5
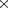
5 neighborhood. There were a total of 625 partially overlapping neighborhoods that were centered around the 625 simple cells. Next, the weights between the input and the simple cells were estimated from 50000 patches of size 32
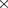
32 pixels by maximizing the sparseness of the locally pooled simple cell energies. Each simple cell was fully connected to the input (i.e. patch of size 32
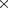
32 pixels). The patches were randomly sampled from the 1750 images of size 128
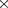
128 pixels in the estimation set. To maximize the sparseness, the energy function (i.e. square nonlinearity) encourages the simple cell responses to be similar within the neighborhoods while the sparsity function (i.e. convex nonlinearity) encourages the locally pooled simple cell energies to be thinly dispersed across the neighborhoods. As a result, the simple cells that are in the same neighborhood have simultaneous activation and similar preferred parameters. Since the neighborhoods overlap, the preferred parameters of the simple and complex cells change smoothly across the grid graph. Finally, the complex cell responses of the SC model were defined as a static nonlinear function of the locally pooled simple cell energies after model estimation (i.e. total of 625 complex cell responses per patch of size 32
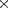
32 pixels and 10000 complex cell responses per image of size 128
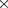
128 pixels). The SC model learned topographically organized, spatially localized, oriented and bandpass simple and complex cell receptive fields that were similar to those found in the primary visual cortex (Figure 2A) [23]–.

To establish a baseline, we used a GWP model [25], [27], [28] of 10921 phase-invariant complex cells [8]. Variants of this model were used in a series of seminal encoding and decoding studies [8], [13], [14], [16]. Note that the fMRI data set was the same as that in [8], [13]. Concretely, the GWP model was a hand-designed population of quadrature-phase Gabor wavelets that spanned a range of locations, orientations and spatial frequencies (Figure 2B). Each wavelet was fully connected to the input (i.e. image of size 128
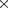
128 pixels). The complex cell responses of the GWP model were defined as a static nonlinear function of the pooled energies of the quadrature-phase wavelets that had the same location, orientation and spatial frequency (i.e. total of 10921 complex cell responses per image of size 128
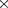
128 pixels).

### Voxel models

To learn the voxel transformation, we used regularized linear regression. The voxel models were estimated from the 1750 feature-transformed stimulus-response pairs in the estimation set by minimizing the 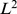 penalized least squares loss function. The combination of a voxel model with the complex cells of the SC and GWP models resulted in two encoding models (i.e. SC2 and GWP2 models). The SC2 model linearly pooled the 10000 complex cell responses of the SC model. The GWP2 model linearly pooled the 10921 complex cell responses of the GWP model.

### Receptive fields

**Figure 3:**
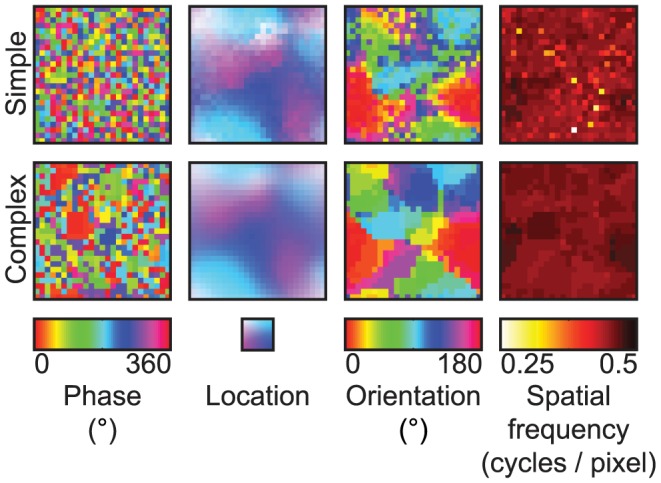
Preferred parameter maps of the SC model. The phase, location, orientation and spatial frequency preference of the simple and complex cells were quantified as the corresponding parameters of Gabor wavelets that were fit to their receptive fields. Each pixel in a parameter map shows the corresponding preferred parameter of a simple or complex cell. The adjacent simple and complex cells had similar location, orientation and spatial frequency preference but different phase preference.

**Figure 4:**
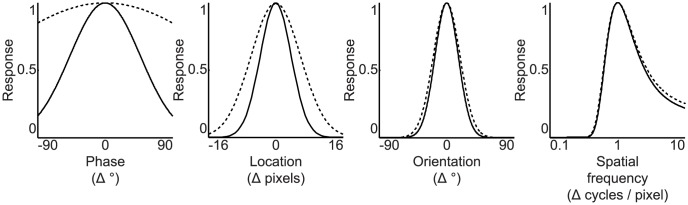
Population parameter tuning curves of the SC model. The population phase, location, orientation and spatial frequency tunings of the simple (solid lines) and complex cells (dashed lines) were quantified by fitting Gaussian functions to the median of their responses to Gabor wavelets that had different parameters. Each curve shows the median of their responses as a function of change in their preferred parameter. The complex cells were more invariant to phase and location than the simple cells.

We first analyzed the receptive fields of the SC model (i.e. simple and complex cell receptive fields). The preferred phase, location, orientation and spatial frequency of the simple and complex cells were quantified as the corresponding parameters of Gabor wavelets that were fit to their receptive fields. The preferred parameter maps of the simple and complex cells were constructed by arranging their preferred parameters on the grid graph (Figure 3). Most adjacent simple and complex cells had similar location, orientation and spatial frequency preference, whereas they had different phase preference. In agreement with [22], the preferred phase, location and orientation maps reproduced some of the salient features of the columnar organization of the primary visual cortex such as lack of spatial structure [29], retinotopy [30] and pinwheels [31], respectively. In contrast to [22], the preferred spatial frequency maps failed to reproduce cytochrome oxidase blobs [32]. The preferred phase map of the simple cells suggests that the complex cells are more invariant to phase and location than the simple cells since the complex cells pooled the energies of the simple cells that had different phase preference. To verify the invariance that is suggested by the preferred phase map of the simple cells, the population parameter tuning curves of the simple and complex cells were constructed by fitting Gaussian functions to the median of their responses to Gabor wavelets that had different parameters (Figure 4). Like the simple cells, most complex cells were selective to orientation (i.e. standard deviation of 21.8° versus 22.9°) and spatial frequency (i.e. standard deviation of 0.52 versus 0.54 in normalized units). Unlike the simple cells, most complex cells were more invariant to phase (i.e. standard deviation of 50.0° versus 158.1°) and location (i.e. standard deviation of 3.70 pixels versus 5.86 pixels). Therefore, they optimally responded to Gabor wavelets that had a specific orientation and spatial frequency, regardless of their phase and exact position.

**Figure 5:**
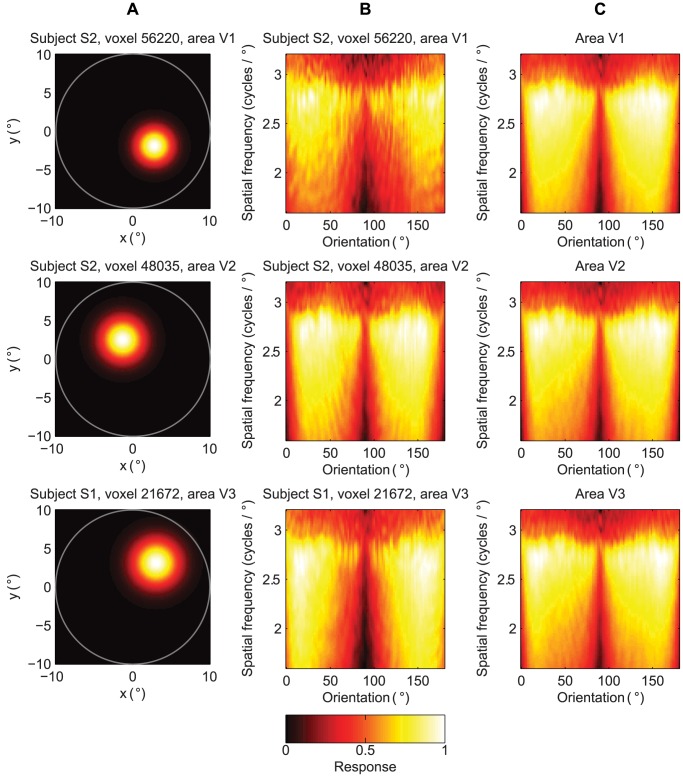
Receptive fields of the SC2 model. The parameter tuning varied across the voxels and had a bias for high spatial frequencies and oblique orientations. (A) Two-dimensional Gaussian functions that were fit to the responses of three representative voxels to point stimuli at different locations. (B) Responses of three representative voxels to sine-wave gratings that spanned a range of orientations and spatial frequencies. (C) Mean responses across the voxels to sine-wave gratings that spanned a range of orientations and spatial frequencies.

**Figure 6:**
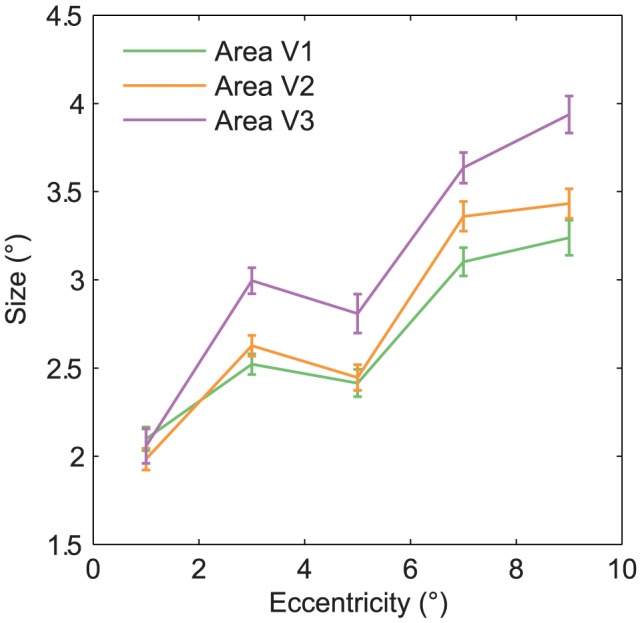
Receptive field size of the SC2 model as a function of receptive field eccentricity of the SC2 model and area. The eccentricity and size of the receptive fields were quantified as the mean and standard deviation of two-dimensional Gaussian functions that were fit to the voxel responses to point stimuli at different locations, respectively. The receptive field size systematically increased from low to high receptive field eccentricity and from area V1 to V3. Error bars show 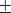
1 SEM across the voxels (bootstrapping method).

We then analyzed the receptive fields of the SC2 model (i.e. voxel receptive fields). The eccentricity and size of the receptive fields were quantified as the mean and standard deviation of two-dimensional Gaussian functions that were fit to the voxel responses to point stimuli at different locations, respectively. The orientation and spatial frequency tuning of the receptive fields were taken to be the voxel responses to sine-wave gratings that spanned a range of orientations and spatial frequencies. While the eccentricity, size and orientation tuning varied across voxels, most voxels were tuned to relatively high spatial frequencies (Figure 5A and Figure 5B). The mean predicted voxel responses to sine-wave gratings that had oblique orientations were higher than those that had cardinal orientations and this difference decreased with spatial frequency (Figure 5C). While this result is in contrast to those of the majority of previous single-unit recording and fMRI studies [33], [34], it is in agreement with those of [35]. In line with [36], [37], the receptive field size systematically increased from V1 to V3 and from low receptive field eccentricity to high receptive field eccentricity (Figure 6). The properties of the GWP2 model were similar to those in [8]. The relationship between the receptive field parameters (i.e. size, eccentricity, area) of the GWP2 model were the same as those of the SC2 model. However, the GWP2 model did not have a large orientation bias.

### Encoding

**Figure 7:**
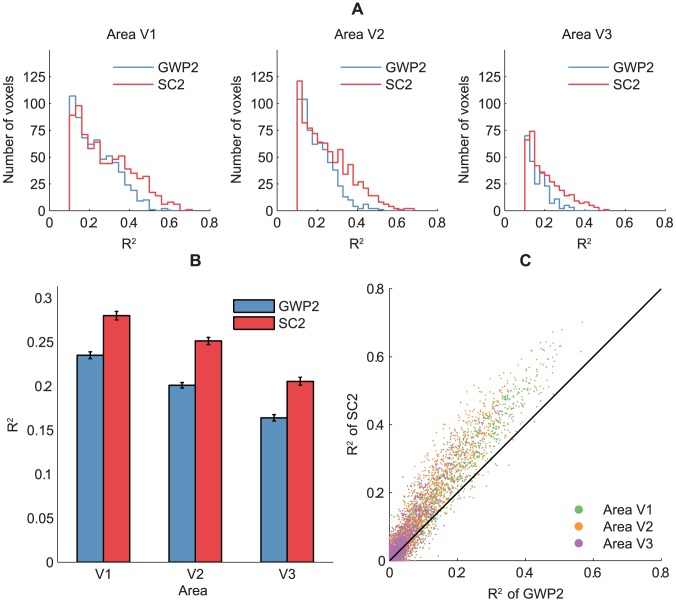
Encoding performance of the SC2 and GWP2 models. The encoding performance was defined as 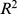 between the observed and predicted voxel responses to the 120 images in the validation set across the two subjects. The encoding performance of the SC2 model was significantly higher than that of the GWP2 model. (A) Prediction 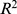 across the voxels that survived the 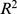 threshold of 0.1. (B) Mean prediction 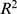 across the voxels that survived the 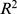 threshold of 0.1. Error bars show 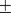
1 SEM across the voxels (bootstrapping method). (C) Prediction 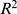 in each voxel.

The encoding performance of the SC2 and GWP2 models was defined as the coefficient of determination (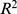) between the observed and predicted voxel responses to the 120 images in the validation set across the two subjects. The performance of the SC2 model was found to be significantly higher than that of the GWP2 model (binomial test, 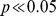). Figures 7A and 7B compare the performance of the models across the voxels that survived an 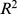 threshold of 0.1. The mean 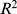 of the SC2 model systematically decreased from 0.28 across 28% of the voxels in V1 to 0.21 across 11% of the voxels in V3. In contrast, the mean 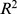 of the GWP2 model systematically decreased from 0.24 across 24% of the voxels in V1 to 0.16 across 6% of the voxels in V3. Figure 7C compares the performance of the models in each voxel. More than 71% of the voxels that did not survive the threshold in each area and more than 92% of the voxels that survived the threshold in each area were better predicted by the SC2 model than the GWP2 model. These results suggest that statistically adapted low-level sparse representations of natural images better span the space of early visual cortical representations than the Gabor wavelets.

### Decoding

**Figure 8:**
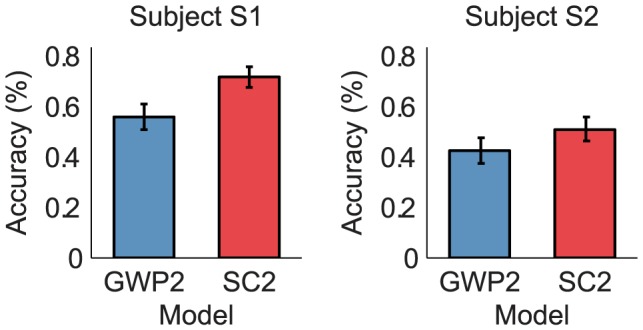
Decoding performance of the SC2 and GWP2 models. The decoding performance was defined as the accuracy of identifying the 120 images in the validation set from a set of 9264 candidate images. The decoding performance of the SC2 model was significantly higher than that of the GWP2 model. Error bars show 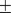
1 SEM across the images in the validation set (bootstrapping method). A more detailed figure that shows the identified images is provided at http://www.ccnlab.net/research/.

The decoding performance of the SC2 and GWP2 models was defined as the accuracy of identifying the 120 images in the validation set from a set of 9264 candidate images. The set of candidate images contained the 120 images in the validation set and the 9144 images in the Caltech 101 data set [38]. Note that the set of candidate images was ten- to hundred-fold larger than the sets in [8] but comparable to the largest set in [15]. The performance of the SC2 model was found to be significantly higher than that of the GWP2 model (binomial test, 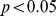). Figure 8 compares the performance of the models. The mean accuracy of the SC2 model across the subjects was 61%. In contrast, the mean accuracy of the GWP2 model across the subjects was 49%. The chance-level accuracy was 0.01%. These results suggest that statistically adapted low-level sparse representations of natural images can be more effectively exploited in stimulus identification than the Gabor wavelets.

### Spatial invariance

**Figure 9:**
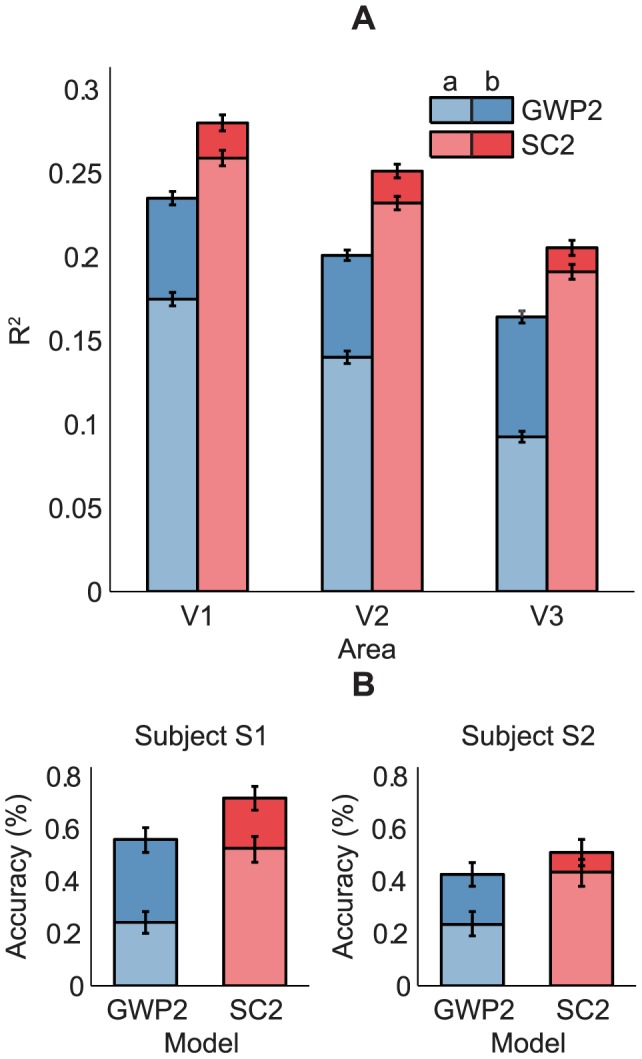
Mean prediction 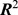 and identification accuracy of the SC2 and GWP2 models after (a) and before (b) translating the images in the validation set by 0.8° in a random dimension. The SC2 model was more invariant than the GWP2 model and its invariance increased from V1 to V3. (A) Mean prediction 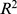 across the voxels that survived the 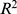 threshold of 0.1 in the case of (b). Error bars show 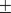
1 SEM across the voxels (bootstrapping method). (B) Identification accuracy. Error bars show 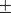
1 SEM across the images in the validation set (bootstrapping method).

In principle, the SC2 and GWP2 models should have some degree of spatial invariance since they linearly pooled the responses of the complex cells that displayed insensitivity to local stimulus position. Spatial invariance is of particular importance for decoding since a reliable decoder should be able to identify a stimulus, regardless of its exact position. Furthermore, a difference between the degree of spatial invariance of the models can be a contributing factor to the difference between their performance. To analyze the spatial invariance of the models, we evaluated their encoding and decoding performance after translating the images in the validation set by 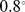 (i.e. approximately the standard deviation of the population location tuning curves of the complex cells of the SC model) in a random dimension (Figure 9). The encoding and decoding performance of the models was found to decrease after the translations. Unlike the encoding performance of the GWP2 model, that of the SC2 model decreased less in V3 than V1. This result suggests greater spatial invariance in V3 than V1. The difference between the mean 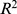 of the models across the voxels that survived the threshold before the translations increased from 0.05 to 0.11. The difference between the mean accuracy of the models across the subjects increased from 12% to 24%. These results suggest that the SC2 model is more tolerant to local translations in stimulus position than the GWP2 model.

### Control models

**Figure 10:**
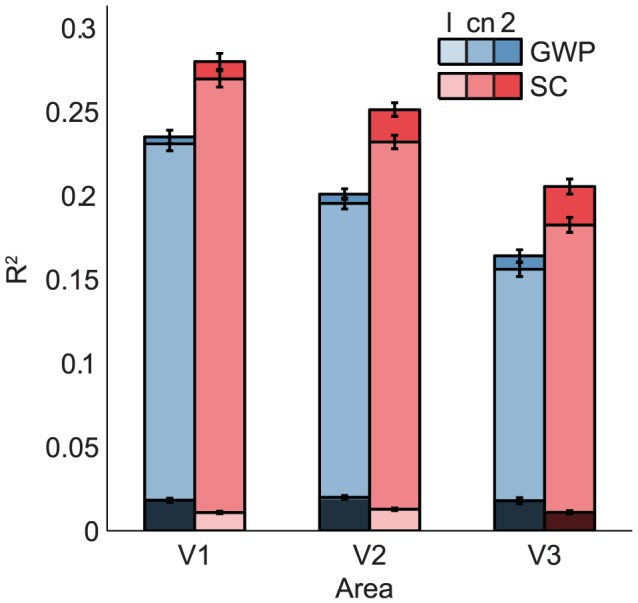
Mean prediction 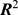 of the linear one-layer (l), compressive nonlinear one-layer (cn) and nonlinear two-layer (2) SC and GWP models across the voxels that survived the 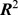 threshold of 0.1 in the case of (2). The mean prediction 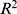 of the linear one-layer models were below the 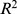 threshold of 0.1. The mean prediction 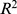 of the nonlinear SC models were significantly better than those of the nonlinear GWP models. The compressive nonlinearity and the nonlinear second layer increased the mean prediction 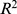 of the linear and compressive nonlinear models, respectively. The nonlinear second layer increased the mean prediction 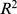 of the compressive nonlinear SC model more than it increased that of the compressive nonlinear GWP model. The error bars show 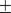
1 SEM across the voxels (bootstrapping method).

Since the SC2 and GWP2 models had different nonlinearities (i.e. pooling and static nonlinearity), a direct evaluation of the contribution of their components (i.e. representations and nonlinearities) to the difference between their encoding performance was not possible. Therefore, we estimated two control models that pooled the same static nonlinear function of the simple cell responses of the SC and GWP models. The static nonlinear function was a compressive nonlinearity (i.e. 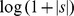 where 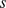 is a simple cell response). The compressive nonlinearity roughly accounts for insensitivities by increasing responses to a stimulus that is not entirely within a receptive field [39]. The simple cell responses were defined as the linear responses of the first layer of the SC model and the even-symmetric Gabor wavelets. While the performance of the compressive nonlinear SC model was significantly higher than that of the compressive nonlinear GWP model, the difference between the performance of the compressive nonlinear models was significantly lower than that of the SC2 and GWP2 models (Figure 10). This result suggests that both the representations and the nonlinearities of the SC2 model contribute to the difference between the encoding performance of the SC2 and GWP2 models.

To verify the contribution of the nonlinearities to the individual encoding performance of the SC2 and GWP2 models, we estimated two more control models that pooled a linear function of the simple cell responses of the SC and GWP models. We used linear models since they retain selectivities that are discarded by nonlinearities. We found that the performance of the linear models were significantly lower than that of the compressive nonlinear, SC2 and GWP2 models (Figure 10). This result confirms the contribution of the nonlinearities that introduced the insensitivities to the individual encoding performance of the SC2 and GWP2 models.

## Discussion

This study addresses the question of how to model feature spaces to better predict brain activity. We introduced a general approach for making directly testable predictions of single voxel responses to statistically adapted representations of ecologically valid stimuli. Our approach relies on unsupervised learning of a feature model followed by supervised learning of a voxel model. To benchmark our approach against the conventional approach that makes use of predefined feature spaces, we compared a two-layer sparse coding model of simple and complex cells with a Gabor wavelet pyramid model of phase-invariant complex cells. While the GWP model is the fundamental building block of many state-of-the-art encoding and decoding models, the GWP2 model was found to be significantly outperformed by the SC2 model. We used control models to determine the contribution of the different components of the SC2 and GWP2 models to this performance difference. Analyses revealed that the SC2 model better accounts for both the representations and the nonlinearities of the voxels in the early visual areas than the GWP2 model. Given that the representations of the SC2 model are qualitatively similar to those of the GWP model, their contribution to this performance difference suggests that the SC model automatically learns an optimal set of spatially localized, oriented and bandpass representations that better span the space of early visual cortical representations since it adapts to the same statistical regularities in the environment as the brain is assumed to be adapted to [20].

Our approach eliminates the need for predefining feature spaces. However, the SC model does have a number of free parameters (e.g. patch size, number of simple and complex cells, etc.) that must either be specified by hand or using model selection methods such as cross-validation. Because of computational considerations, we used the same free parameters as those in [22]. While the choice of these free parameters can influence what the SC model can learn, the SC2 model was shown to outperform the GWP2 model even without cross-validation. Next to cross-validation, other methods that also infer these free parameters can further improve the performance of the SC2 model. One method is to first estimate voxel receptive fields using any approach and then use these estimates as free parameters (e.g. voxel receptive field eccentricity as patch size) of voxel-specific feature models. Another method is to use more sophisticated nonparametric Bayesian sparse factor models [40] that can simultaneously learn sparse representations while inferring their number. Furthermore, our approach included only feedforward projections such that representations and responses were solely determined by stimuli. However, taking top-down modulatory effects into account is essential to adequately characterize how sensory information is represented and processed in the brain. For example, attention has been shown to warp semantic representations across the human brain [41], and prior expectations have been shown to bias sensory representations in visual cortex [42]. Extensions of our approach that include feedback projections can be used to address the question of how representations and responses are influenced by top-down processes.

Further extensions of our approach can be used to probe mid- to high-level extrastriate visual cortical representations in a fully automated manner. In particular, the SC model can be replaced by highly nonlinear multi-layer statistical models of natural images that learn hierarchical feature spaces (i.e. deep learning [43]). Some of the feature spaces that are learned by these models such as mid-level edge junctions have been shown to match well with neural response functions in area V2 [44]. Models that learn even higher-level representations such as high-level object parts [45] or complete objects [46] can be used to probe extrastriate visual cortical representations. For example, heterogenous hierarchical convolutional neural networks have been shown to predict the representational dissimilarity matrices that characterize representations in human inferior temporal gyrus [47]. Similar models have been shown to learn feature spaces that are admitted by stimulus sets other than natural images, both within the visual modality (e.g. natural movies [48]) as well as in other modalities (e.g. auditory or somatosensory [49]). These models can be used to probe cortical representations in different sensory modalities.

One approach to estimate deep models is to maximize the likelihood of all layers at the same time. However, this approach is not scalable and requires the computation of intractable partition functions that are impossible to integrate analytically and computationally expensive to integrate numerically. Nevertheless, methods such as score-matching [50] and noise-contrastive estimation [51] have been used to estimate unnormalized nonlinear multi-layer statistical models of natural images [52], [53]. An alternative approach is to use models such as deep belief networks that comprise multiple layers of restricted Boltzmann machines. These models can be scaled by convolution [45] and estimated by maximizing the likelihood of one layer at a time, using the output of each layer as input for the subsequent layer [54]. Importantly, generative models such as deep belief networks make it possible to sample stimuli based on internal network states. Conditioning these internal network states on stimulus-evoked brain activity results in a generative approach to decoding. For example, we have previously shown that a deep belief network that comprise multiple layers of conditional restricted Boltzmann machines can reconstruct handwritten digits by sampling from the model after conditioning it on stimulus-evoked multiple voxel responses [55].

While introducing a new approach to probe cortical representations, this study complements other developments in encoding and decoding. For example, encoding models that involve computations to account for contrast saturation or heterogeneous contrast energy were shown to improve prediction of single voxel responses to visual stimuli [16]. At the same time, these modeling efforts go hand in hand with developments in fMRI such as the improvements in contrast-to-noise ratio and spatial resolution that are facilitated by increases in magnetic field strength [56]. For example, spatial features of orientation-selective columns in humans were demonstrated by using high-field fMRI [57]. Jointly, such developments can provide novel insights into how cortical representations are learned, encoded and transformed.

In conclusion, we introduced a general approach that improves prediction of human brain activity in response to natural images. Our approach primarily relies on unsupervised learning of transformations of raw stimuli to representations that span the space of cortical representations. These representations can also be effectively exploited in stimulus classification, identification or reconstruction. Taken together, unsupervised feature learning heralds new ways to characterize the relationship between stimulus features and human brain activity.

## Materials and Methods

### Data

We used the fMRI data set [21] that was originally published in [8], [13]. Briefly, the data set contained 1750 and 120 stimulus-response pairs of two subjects (i.e. S1 and S2) in the estimation and validation sets, respectively. The stimulus-response pairs consisted of grayscale natural images of size 128
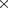
128 pixels and stimulus-evoked peak BOLD hemodynamic responses of 5512 (S1) and 5275 (S2) voxels in the early visual areas (i.e. V1, V2 and V3). The details of the experimental procedures are presented in [8].

### Problem statement

#### Encoding

Let 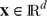 and 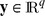 be a stimulus-response pair where 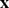 is a vector of pixels in a grayscale natural image, and 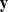 is a vector of voxel responses. The parameters 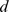 and 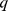 denote the number of pixels and voxels, respectively. Given 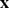, we are interested in the problem of predicting 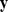: 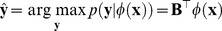
(1)where 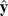 is the predicted response to 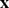, and 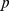 is the encoding distribution of 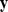 given 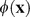. The function 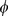 nonlinearly transforms 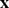 from the stimulus space to the feature space, and 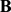 linearly transforms 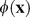 from the feature space to the voxel space.

#### Decoding

Let 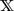 be a set of images that contains 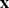. Given 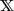 and 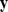, we are interested in the problem of identifying 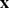: 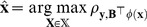
(2)where 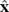 is the identified image from 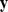, and 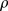 is the Pearson product-moment correlation coefficient between 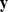 and 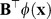.

Solving the encoding and decoding problems requires the definition and estimation of a feature model 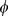 followed by a voxel model 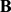.

### Feature model

#### Model definition

Following [22], we summarize the definition of the SC model. We start by defining a single-layer statistical generative model of whitened grayscale natural image patches. Assuming that a patch is generated by a linear superposition of latent variables that are non-Gaussian (in particular, sparse) and mutually independent, we first use independent component analysis to define the model by a linear transformation of independent components of the patch: 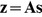
(3)where 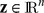 is a vector of pixels in the patch, 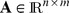 is a mixing matrix, and 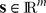 is a vector of the components of 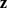 such that 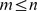. The parameters 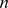 and 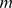 denote the number of pixels and components, respectively. We then define 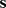 by inverting the linear system that is defined by 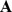:
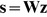
(4)where 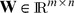 is an unmixing matrix such that 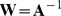. We constrain 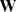 to be orthonormal and 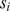 to have unit variance such that 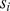 are uncorrelated and unique, up to a multiplicative sign. Next, we define the joint probability of 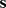 by the product of the marginal probabilities of 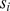 since 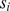 are assumed to be independent:
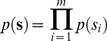
(5)where 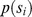 are peaked at zero and have high kurtosis since 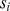 are assumed to be sparse.

While one of the assumptions of the model is that 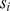 are independent, their estimates are only maximally independent. As a result, residual dependencies remain between the estimates of 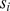. We continue by modeling the nonlinear correlations of 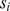 since 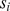 are constrained to be linearly uncorrelated. In particular, we assume that the locally pooled energies of 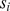 are sparse. Without loss of generality, we first arrange 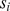 on a square grid graph that has circular boundary conditions. We then define the locally pooled energies of 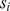 by the sum of the energies of 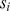 that are in the same neighborhood: 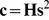
(6)where 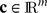 is a vector of the locally pooled energies of 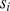 and 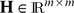 is a neighborhood matrix such that 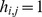 if 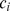 pools the energy of 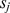 and 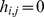 otherwise. Next, we redefine 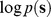 in terms of 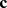 to model both layers:
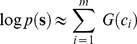
(7)where 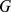 is a convex function. Concretely, we use 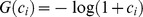.

In a neural interpretation, simple and complex cell responses can be defined as 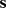 and a static nonlinear function of 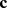, respectively. Concretely, we use 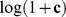 to define the complex cell responses after we estimate the model.

#### Model estimation

We use a modified gradient ascent method to estimate the model by maximizing the log-likelihood of 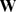 (equivalently, the sparseness of 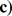 given a set of patches: 
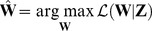
(8)where 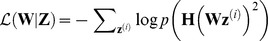 is an approximation of the log-likelihood of 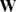 and 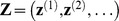 is the set of patches. At each iteration, we first find the gradient of 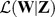:
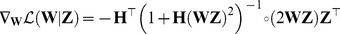
(9)where 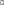 is the Hadamard (element-wise) product. We then project it onto the tangent space of the constrained space [58]:
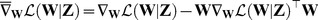
(10)Next, we use backtracking line search to choose a step size by reducing it geometrically with a rate from 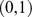 until the Armijo-Goldstein condition holds [59]. Finally, we update 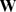 and find its nearest orthogonal matrix: 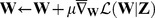
(11)

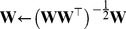
(12)where 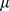 is the step size.

### Voxel model

#### Model definition

We start by defining a model for each voxel. Assuming that 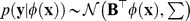, where 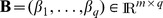 and 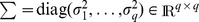, we use linear regression to define the models by a weighted sum of 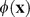: 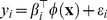
(13)where 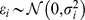.

#### Model estimation

We estimate the model using ridge regression: 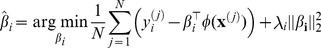
(14)where 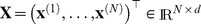 and 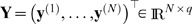 is an estimation set, and 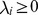 is a complexity parameter that controls the amount of regularization. The parameter 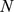 denotes the number of stimulus-response pairs in the estimation set. We obtain 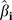 as:
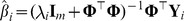
(15)where 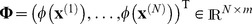 and 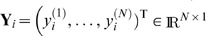. Since 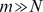, we solve the problem in a rotated coordinate system in which only the first N coordinates of 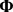 are nonzero [60], [61]. We first factorize 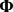 using the singular value decomposition:
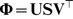
(16)where 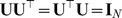, 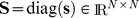 and 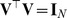. The columns of 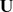, the diagonal entries of 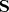 and the columns of 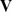 are the left-singular vectors, the singular values and the right-singular vectors of 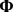, respectively. We then reobtain 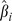 as:
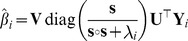
(17)where division is defined element-wise. The rotation reduces the complexity of the problem from 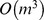 to 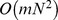. To choose the optimal 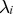, we perform hyperparameter optimization using grid search guided by a generalized cross-validation approximation to leave-one-out cross-validation [60]. We define a grid by first sampling the effective degrees of freedom of the ridge regression fit from 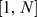 since its parameter space is bounded from above. The effective degrees of freedom of the ridge regression fit is defined as:



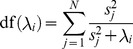
(18)We then use Newton's method to solve 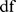 for 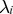. Once the grid is defined, we choose the optimal 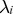 that minimizes the generalized cross-validation error: 
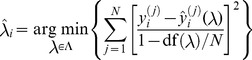
(19)where 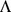 is the grid, and 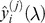 is 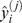 given a particular 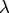.

### Encoding and decoding

In the case of the SC model, each randomly sampled or non-overlapping patch was transformed to its principal components such that 625 components with the largest variance were retained and whitened prior to model estimation and validation. After the images were feature transformed, they were z-scored. The SC model of 625 simple and 625 complex cells was estimated from 50000 patches of size 32
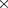
32 pixels that were randomly sampled from the 1750 images of size 128
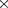
128 pixels in the estimation set. The details of the GWP model are presented in [8]. The SC2 and GWP2 models were estimated from the 1750 feature-transformed stimulus-response pairs in the estimation set.

Voxel responses to an image of size 128
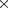
128 pixels were predicted as follows. In the case of the SC model, each 16 non-overlapping patch of size 32
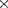
32 pixels of the image were first transformed to the complex cell responses of the SC model (i.e. total of 625 complex cell responses per patch and 10000 complex cell responses per image). The 10000 complex cell responses of the SC model were then transformed to the voxel responses of the SC2 model. In the case of the GWP model, the image was first transformed to the complex cell responses of the GWP model (i.e. total of 10921 complex cell responses per image). The 10921 complex cell responses of the GWP model were then transformed to the voxel responses of the GWP2 model. The encoding performance was defined as the coefficient of determination between the observed and predicted voxel responses to the 120 images in the validation set across the two subjects.

A target image was identified from a set of candidate images as follows. Prior to identification, 500 voxels were selected without using the target image. The selected voxels were those whose responses were predicted best. The target image was identified as the candidate image such that the observed voxel responses to the target image were most correlated with the predicted voxel responses to the candidate image (i.e. highest Pearson product-moment correlation coefficient between observed and predicted voxel responses). The decoding performance was defined as the accuracy of identifying the 120 images in the validation set from the set of 9264 candidate images. The set of candidate images contained the 120 images in the validation set and the 9144 images in the Caltech 101 data set [38].
